# Phylogenetic and Expression Analysis of the Sucrose Synthase and Sucrose Phosphate Synthase Gene Family in Potatoes

**DOI:** 10.3390/metabo14010070

**Published:** 2024-01-20

**Authors:** Jun Hu, Yanfeng Duan, Jinxue Hu, Shuqing Zhang, Guangcun Li

**Affiliations:** 1 State Key Laboratory of Vegetable Biobreeding, Institute of Vegetables and Flowers, Chinese Academy of Agricultural Sciences, Beijing 100081, China; hujun@caas.cn (J.H.);; 2 Key Laboratory of Biology and Genetic Improvement of Tuber and Root Crop, Ministry of Agriculture and Rural Affairs, Institute of Vegetables and Flowers, Chinese Academy of Agricultural Sciences, Beijing 100081, China; 3 Shijiazhuang Academy of Agriculture and Forestry Sciences, Shijiazhuang 050041, China

**Keywords:** *Solanum tuberosum* L., sucrose metabolism, gene expression, bioinformatics analysis

## Abstract

Sucrose synthase (SUS) and sucrose phosphate synthase (SPS) are essential in plant sucrose metabolism. The potato is an important crop worldwide, but systematic analyses of the *StSUS* and *StSPS* gene families in potatoes are still lacking. Ten sucrose metabolism-related genes were identified in this study. The *SUSs* and *SPSs* could each be split into three subgroups through phylogenetic analysis. *StSUSIc* was the most highly expressed gene in different developmental tissues. Ka/Ks analysis showed that *StSUSIb* and *StSUSIc* were subjected to more-significant homozygous selection pressure. Our cis-acting element analysis of the *StSUS* and *StSPS* promoter sequences showed four elements: defense- and stress-responsive, hormone-responsive, light-responsive, and transcription factor elements. The expression of *StSUS* and *StSPS* genes was found to be regulated by circadian rhythm. In the treatments of 1% to 5% sucrose, glucose, and fructose, the expression of *StSUS* and *StSPS* family genes was enhanced by sucrose, but inhibited at high-glucose and fructose concentrations. This study identified six *StSUS* and four *StSPS* genes and analyzed their gene structure, conserved motifs, chromosome position, promoter elements, phylogenetic tree, and tissue-specific expression patterns. Our results will motivate more research into the biological process underlying the genes of sucrose metabolism in potatoes.

## 1. Introduction

Sucrose synthesized by photosynthesis is continuously transported to the active sink organ with anabolism through the phloem by sucrose transporters, such as fruits, seeds, and tubers. Sucrose is then converted into fructose, and glucose accumulates in the vacuoles of storage cells. Sucrose level signaling also regulates multiple developmental processes in plants, including cell division, ribosome synthesis, cotyledon, tuber development, anthocyanin accumulation, and floral organ induction [1,2]. 

Sucrose synthase (SUS) is vital for the sucrose metabolism pathway in plants. SUS is thought to break down sucrose to produce adenosine diphosphate glucose (ADPG) in plants [3]. Inhibiting *SUS* gene expression in potatoes resulted in a decrease in SUS activity in tubers and no significant changes in the other enzymes involved in starch synthesis; however, there was a decrease in starch content in the tubers, with uridine diphosphate glucose (UDPG) and ADPG levels only reaching 30% and 35%, respectively, of the control levels [4]. Overexpression of the *SUS* gene in potatoes enhanced the UDPG and ADPG content, tuber starch content, and total tuber yield [5]. Potato *StSUS4* was transformed into maize in order to enhance SUS activity, ADPG levels, and starch content in transgenic maize seeds [6]. In a study of *Arabidopsis thaliana* SUS mutants, the SUS activity in the leaves and stems of the mutant plants was 85% of that of the wild-type leaves [7]. Inhibiting the strawberry (*Fragaria* × *ananassa*) *FaSUS1* gene through RNAi technology delays anthocyanin accumulation and significantly slows strawberry fruit ripening [8]. Sucrose synthase 3 confers high-temperature tolerance at rice maturity [9]. Silencing *StSUSI* via RNAi decreases SUS activity and starch content in tubers [4]. When *StSUS4* was overexpressed in tubers, the contents of UDPG, ADPG, and starch, and the total tuber yield were significantly increased [5]. 

Sucrose phosphate synthase (SPS) is a critical enzyme that controls plant sucrose biosynthesis. SPS catalyzes UDPG and fructose-6-phosphate (F6P) to produce sucrose-6-phosphate (S6P) and uridine diphosphate (UDP). There are four *SPS* genes in *A. thaliana*, cocoa, and litchi. Although the number of *SPS* genes in different species differs, the protein-coding sequences of SPS are relatively conservative and contain three domains: glycosyltransferase, sucrose synthase, and sucrose-6-phosphate phosphohydrolase. In sucrose metabolism, SPS affects the intensity of source and sink organs, regulates photosynthate distribution between sucrose and starch, and participates in cell differentiation, synthesis of the vanguard cell wall, and biomass formation. *NtSPSb* is expressed in all tobacco tissues, and *NtSPSb* participates in sucrose synthesis at night and maintains normal starch transport [10]. Among rice *SPS* genes, *OsSPS1* is preferentially expressed in source organs, while *OsSPS2* and *OsSPS8* are equally expressed in source and sink organs [11]. The *SlSPS* gene regulates tomato growth, development, and heat tolerance [12]. Overexpression of the *SPS* gene increases the biomass and sucrose contents in sugarcane [13]. A recent report has shown that *StSPS1* may regulate seed potato vigor [14].

Multiple aspects, such as light, temperature, moisture, and nutrients, regulate the primary metabolism of plants. The circadian clock is the internal regulation mechanism of plants to respond to day–night rhythms and seasonal changes [15]. The expression of starch synthase genes and sucrose metabolism-related genes in potato leaves and tubers is closely related to starch synthesis metabolism and primary metabolites, and is regulated by rhythm oscillation [16]. *OsSPS1* and *OsSPS11* gene expression is regulated by the circadian rhythm in rice [17]. Sucrose induces hypocotyl elongation in *Arabidopsis*, which is regulated by the expression of the phytochrome-interacting factor, which is dependent on the diurnal pattern of sugars and hormones [18]. Sucrose and ethylene maintain sugar-regulated circadian oscillation in the dark through a post-transcriptional mechanism of the circadian oscillator GIGANTEA (GI) [19]. However, how the expression patterns of *StSUS* and *StSPS* genes are regulated by circadian rhythms is unclear.

The potato (*Solanum tuberosum* L.) is an important crop worldwide. Medium and long days promote its flowering, while short days promote tuber formation. The doubled monoploid *S. phureja* DM1-3 516 R44 (DM) reference genome has been updated with the development of high-throughput sequencing technology [20,21]. In recent years, several tetraploid potato genomes have been sequenced [22,23], and a large number of functional genes and their gene family members have been reported [24,25,26,27]. SUSs and SPSs play crucial roles in sucrose metabolism in plants, but a comprehensive analysis of *StSUS* and *StSPS* gene families in potatoes is still lacking. In this study, six *StSUS* and four *StSPS* genes were isolated from the potato genome and examined to investigate *StSUS* and *StSPS* gene families and their roles in circadian rhythm and sucrose metabolism in potato plants, as well as their gene structure, phylogenetic tree, promoter elements, and expression patterns in response to sucrose signals.

## 2. Materials and Methods

### 2.1. Identifying StSUS and StSPS Family Members in the Potato Genome

The potato genome sequences (*Solanum tuberosum* L.) were downloaded from Phytozome (http://www.phytozome.net/ (accessed on 29 March 2021) and another website (http://www.bioinformaticslab.cn/files/dm8/ (accessed on 25 Augest 2023) to identify the *StSUS* and *StSPS* genes, respectively. *AtSUS* and *AtSPS* aa sequences were downloaded from the TAIR database and were blasted against the potato protein database (e-value < 1*^10^). The resulting proteins were checked using a conserved domain database (CDD) to make sure that the *StSUS* genes contained the PF00862 domain and PF00534 domain, and *StSPS* genes contained the PF05116 domain, PF00862 domain, and PF00534 domain. Finally, six *StSUS* genes and four *StSPS* genes were found in the potato genome. Basic physical and chemical characteristics (such as the aa sequence, molecular weight (Mw), and predicted theoretical isoelectric point (pI)) of the potato StSUS and StSPS proteins were calculated using a ProtParam tool (https://web.expasy.org/protparam/ (accessed on 8 September 2022). Multiple sequence alignments of the aa sequences were performed using DNAMAN7.0 software.

### 2.2. Analysis of Gene Structure and Conserved Motifs

Gene structure was analyzed using Gene Structure Display Server 2.0 (http://gsds.gao-lab.org/ (accessed on 4 July 2022). The conserved motifs and sequences of the StSUS and StSPS proteins were visualized using the Multiple Enrichmment for Motif Elicitation (MEME) program, and the maximum number of motifs was set to 10.

### 2.3. Sequence Alignment and Phylogenetic Analysis

In total, 89 SUS and 88 SPS protein sequences were obtained from 13 species, such as *Arabidopsis thaliana*, *Brassica oleracea*, *Brassica rapa*, *Cucumis sativus*, *Glycine max*, *Malus domestica*, *Musa acuminata*, *Oryza sativa*, *Setaria italica*, *Solanum lycopersicum*, *Solanum tuberosum*, *Triticum aestivum*, and *Zea mays*; these were collected from Phytozome (https://phytozome.jgi.doe.gov/pz/portal.html (accessed on 21 April 2022) and NCBI (www.ncbi.nlm.nih.gov (accessed on 28 April 2022) for alignment and phylogenetic analysis. The protein sequences were aligned using a ClustalW algorithm, and the phylogenetic tree was built using MEGA-X software with neighbor-joining (NJ) and 1000 bootstrap replications [28].

### 2.4. Collinearity and Ka/Ks Analysis

Collinearity relationship analysis of the *StSUS* and *StSPS* gene family in potatoes, *Arabidopsis thaliana*, and tomatoes was conducted using MCScanX [29]. For Ka/Ks calculations, the coding sequences and aa sequences of *StSUSs* and *StSPSs* of the tetraploid Atlantic variety were obtained from the International Potato Genome Sequencing Consortium (PGSC) website (http://spuddb.uga.edu/ (accessed on 19 May 2023) using the blast method, and ClustalX was used for sequence alignment. Ka/Ks calculation software 2.0 [30] was used to estimate the Ka/Ks value using the maximum likelihood estimation method. The ω value was the ratio of non-synonymous substitutions of non-synonymous sites to synonymous substitutions of synonymous sites, and the significance was assessed using a *t*-test.

### 2.5. Analysis of Cis-Acting Elements of StSUS and StSPS Promoters

The promoter sequences were obtained from two thousand bp upstream sequences of the ATG codon of the *StSUS* and *StSPS* genes. PlantCARE online (http://bioinformatics.psb.ugent.be/webtools/plantcare/html/ (accessed on 20 April 2022) was utilized to predict potential cis-acting elements. The results were plotted using TBtools [31].

### 2.6. Analysis of Tissue Gene Expression

Public RNA-seq gene expression data from the PGSC website (http://spuddb.uga.edu/pgsc_download.shtml (accessed on 18 December 2022) were used to analyze the tissue expression levels of the *StSUS* and *StSPS* genes. The gene expression values of the *StSUSs* and *StSPSs* from 10 tissues, including the root, shoot, leaf, flower, tuber, stolon, sepal, petiole callus, petals, stamens, and carpels, were collected from the database. A heat map of each *StSUS* and *StSPS* gene member expression was created using TBtools [31].

### 2.7. Plant Material and Treatment

The tetraploid potato cultivar ‘Desirée’ seedlings were grown on Murashige and Skoog (MS) medium with 3% sucrose and 0.6% agar at a pH of 5.8 at 22 ± 1 °C under long-day conditions (16 h light/8 h dark) at approximately 60–80% humidity. For the treatments with 1%, 3%, and 5% sugar, 1 g, 3 g, and 5 g of sucrose, glucose, and fructose, respectively, were added separately to 100 mL MS medium each, the pH was adjusted to 5.7–5.8, and then they were autoclaved. Samples were collected three weeks after transplantation. Healthy and uniform leaves were randomly collected at 4 h intervals for the circadian rhythm analysis, and three biological replicates were prepared. All samples were collected, immediately frozen in liquid nitrogen, and stored at −80 °C until further analysis.

### 2.8. RNA Extraction, cDNA Synthesis, and qPCR Analysis

Total RNA samples from the potato leaves were extracted using the FastPure Universal Plant Total RNA Isolation Kit (Nanjing Vazyme Biotech Co., Ltd., Nanjing, China), and 0.5–1.0 μg of total RNA was used for cDNA first-strand synthesis using a HiScript III 1st Strand cDNA Synthesis Kit (+gDNA wiper). An SYBR qPCR Master Mix was used for qPCR. The qPCR reactions were performed using a LightCycler 480 machine (Roche, Basel, Switzerland) using the following procedure: 95 °C for 3 min, followed by 45 cycles at 95 °C for 10 s, 60 for 10 s, and 72 °C for 20 s. Each reaction had three replicates. The relative expression levels of the target genes were calculated using the 2^−ΔΔCt^ method. All primers used for the qPCR assay are shown in Table S1. The housekeeping gene *ELF3e* was used as a control.

### 2.9. Statistical Analysis

Statistical analyses were performed using the software in Microsoft Excel 2010 and R 3.4.3 software. The data were statistically evaluated using analyses of variance and Duncan’s test at a *p* < 0.05 significance level. The significant differences are indicated by different letters above the columns in the figures. 

## 3. Results

### 3.1. Identification of StSUS and StSPS Gene Family Members in Potatoes

**Figure 1:**
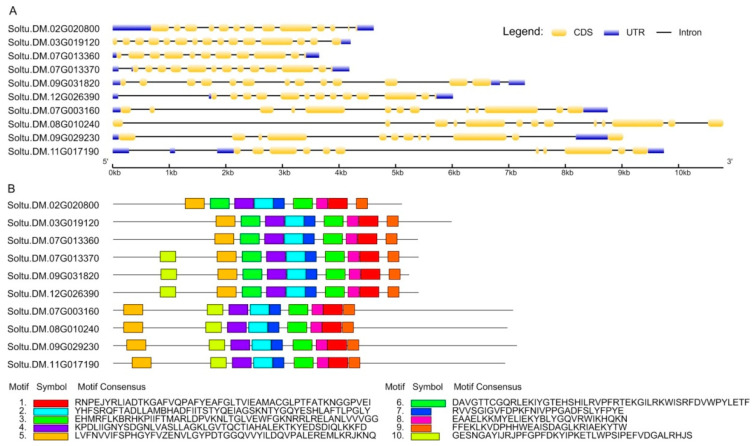
Characterizing 10 identified *StSUSs* and *StSPSs* in the potato genome. (**A**) Gene structures of *StSUS* and *StSPS* genes. Yellow represents exon, blue represents UTR, and the black line represents intron. The scale at the bottom shows the lengths of the sequences. (**B**) Distribution of conserved motifs of the StSUS and *StSPS* proteins. Ten boxes exhibited different conserved motifs, showing the conserved aa sequences.

The potato genome database identified six *StSUS* and four *StSPS* gene family members. The *StSUS* and *StSPS* coding sequence (CDS) length was 2286 to 3195 bp. The amino acid (aa) sequences of the StSUS and StSPS proteins ranged from 761 aa to 1064 aa. Finally, the molecular weight (Mw) of the predicted proteins ranged from 86.7 kDa to 119.6 kDa. The theoretical pI of the *StSUSs* and *StSPSs* ranged from 5.87 to 8.53. All genes encoded acidic proteins except for *StSUSIIIa*. The *StSUS* and *StSPS* genes have 11–15 exons and 10–14 introns, which are similar. Still, the intron length of the *StSPSs* was significantly longer than that of the *StSUSs* (Figure 1A). Among the *StSUS* family, the *StSUS* family genes show 54.1–91.5% sequence similarity at the aa level. The *StSUSIb* and *StSUSIc* aa sequences had the highest similarity. The members of the *StSPS* gene family showed 54.9–70.8% sequence similarity at the aa level. The aa sequences of *StSPSI* and *StSPSII* had the highest similarity. 

The physical locations of *StSUSs* and *StSPSs* were found among seven potato chromosomes (e.g., Chr2, Chr3, Chr7, Chr8, Chr9, Chr11, and Chr12) (Table 1). There were 10 conserved motifs in the *StSUSs* and *StSPSs* (Figure 1B). The motif sequences of different genes were basically the same. Compared with the *StSUSs*, all four *StSPSs* lacked motif 6, a *StSUS*-specific motif. Motifs 5 and 10 exchanged relative positional orders between the *StSUSs* and *StSPSs*.

### 3.2. Phylogenetic Tree Analysis of the Gene Family

**Figure 2:**
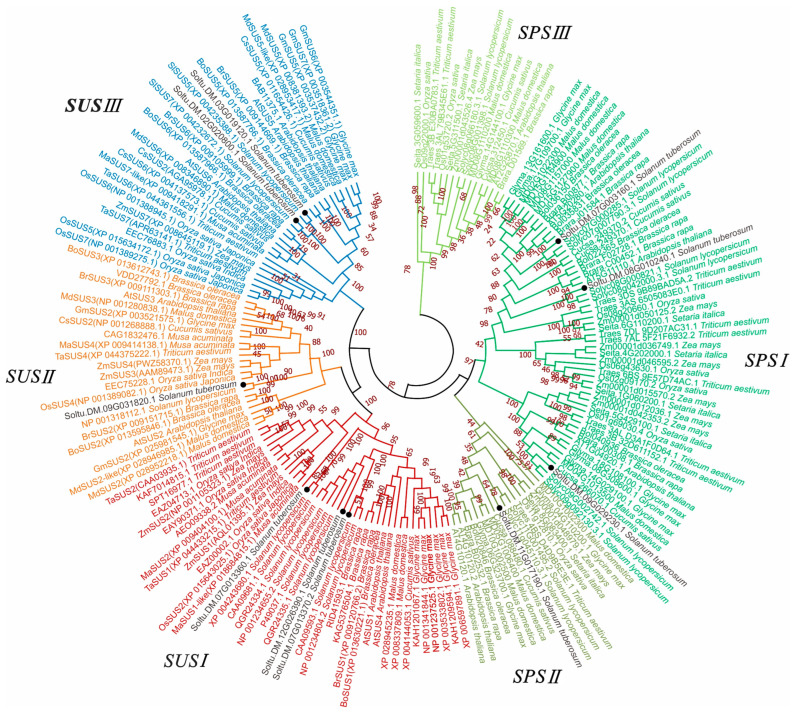
Phylogenetic tree analysis of StSUSs and StSPSs. Protein sequences of SUSs and SPSs from *Arabidopsis thaliana*, *Brassica oleracea*, *Brassica rapa*, *Cucumis sativus*, *Glycine max*, *Malus domestica*, *Musa acuminata*, *Oryza sativa*, *Setaria italica*, *Solanum lycopersicum*, *Solanum tuberosum*, *Triticum aestivum*, and *Zea mays* were collected from Phytozome and NCBI. The phylogenetic tree was constructed using MEG-X software, using the neighbor-joining algorithm with 1000 bootstrap replicates. Red represents *SUSI* group, orange represent *SUSII* group, and blue represent *SUSIII* group. Green represents *SPSI* group, dark green represents *SPSII* group, light green represents *SPSIII* group.

**Figure 3:**
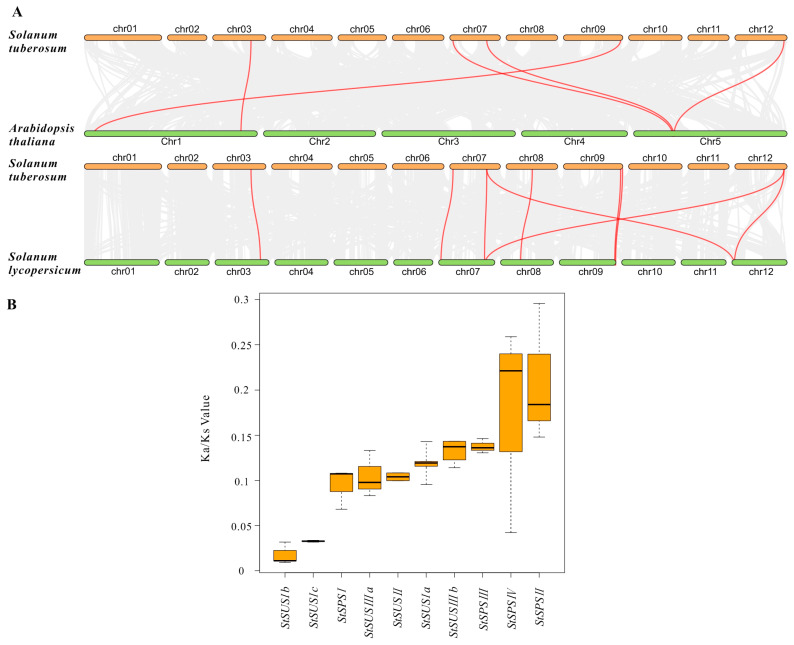
Synteny and Ka/Ks analysis of *StSUSs* and *StSPSs*. (**A**): Visual display of collinearity for *StSUS* and *StSPS* members from *Arabidopsis thaliana*, *Solanum lycopersicum*, *Solanum tuberosum*. (**B**): Comparison of the Ka/Ks ratios of *StSUS* and *StSPS* gene pairs in doubled monoploid potatoes DM 1–3 516 R44 and tetraploid potatoes of the Atlantic variety from the PGSC website.

To understand the evolutionary relationship between *StSUSs* and *StSPSs*, a phylogenetic tree was constructed using 177 homologous proteins from 13 species via the neighbor-joining method. The *SUSs* genes could be divided into three subgroups (Figure 2): *SUSI* had three *StSUSI* genes, *SUSIII* had two *StSUSIII* genes, and *SUSII* had only one *StSUSII*. The *SPSs* could be divided into three subgroups: *SPSI, SPSII*, and *SPSIII*. *SPSI* had three genes (Figure 2). The five genes in the *StSUS* and *StSPS* gene family were collinear with those in the *Arabidopsis* genome. Compared with the tomato genome, nine genes showed collinearity, consistent with expectations (Figure 3A). The Ka/Ks ratio of the *StSUSs* was significantly lower than that of the *StSPSs*, especially *StSUSIb* (0.017) and *StSUSIc* (0.032), which indicated that they were subjected to stronger homozygous selection pressure than other members in the *StSUS* and *StSPS* family (Figure 3B). 

### 3.3. Cis-Acting Element Analysis

**Figure 4:**
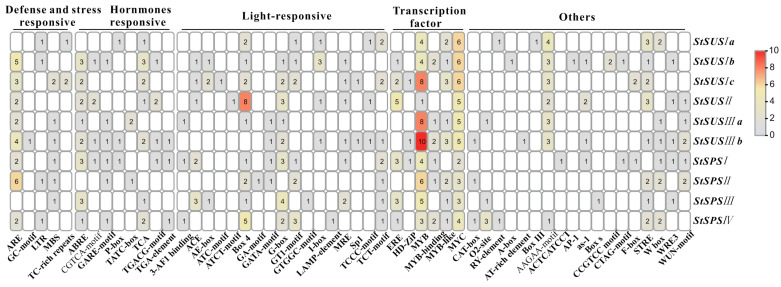
Analysis of the cis-regulatory elements in the promoters of *StSUS* and *StSPS* genes. Five types of cis-elements were found: defense- and stress-responsive, hormone-responsive, light-responsive, transcription factors, and other elements. The values indicate the number of cis-acting elements contained in each promoter sequence.

In analyzing the upstream 2000 bp promoter sequence of *StSUSs* and *StSPSs*, there were a large number of cis-acting elements. Anaerobic response element (ARE) was the most common motif in defense- and stress-responsive elements. The most light-responsive element was Box 4. The number of *v-Myb* myeloblastosis viral oncongene homolog (MYB) transcription factor binding elements was the highest among them, and there were significant differences between the different *StSUSs* and *StSPSs*. There were more cis-acting elements in the *StSUS* promoter than in the *StSPS* promoters (Figure 4). 

### 3.4. Gene Expression of StSUSs and StSPSs in Different Tissues and Circadian Oscillation

**Figure 5:**
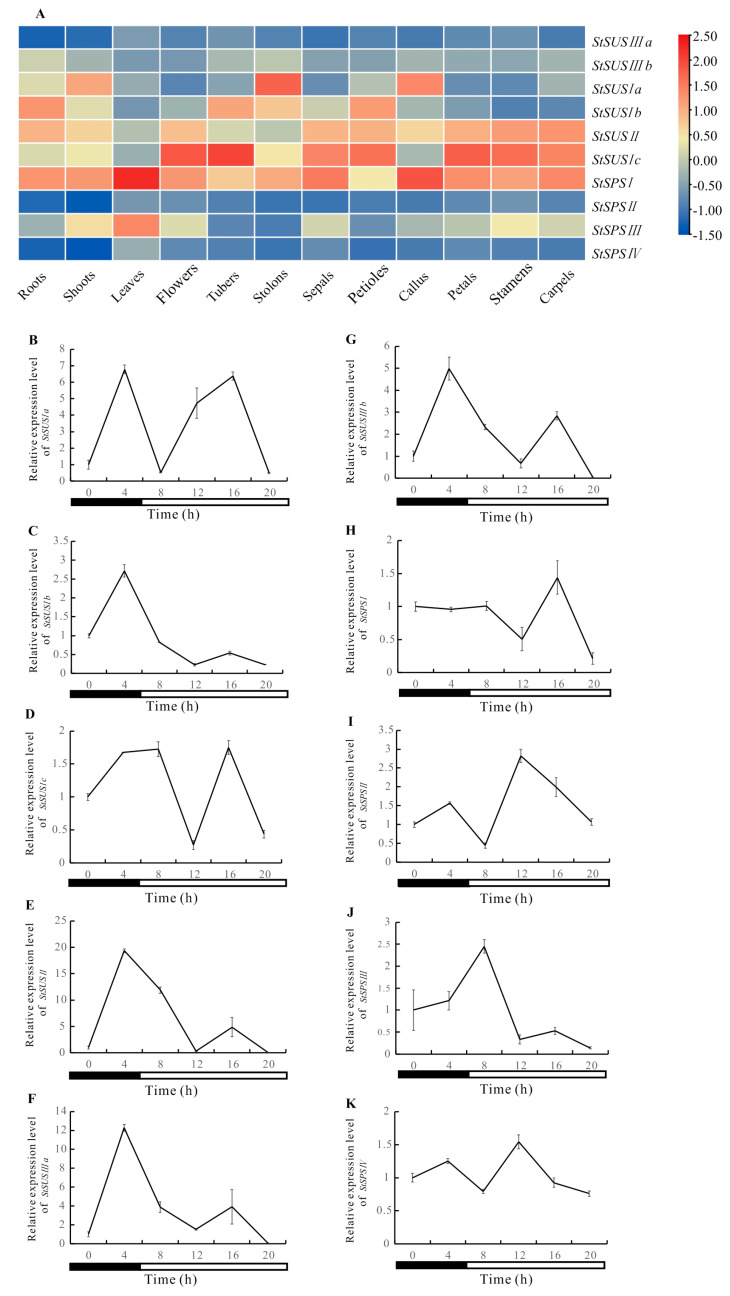
Relative expression of *StSUS* and *StSPS* genes in various tissues and circadian rhythms. (**A**): Heat map of the expression profiles of *StSUS* and *StSPS* genes in various tissues, plotted using TBtools. B–K: Relative expression levels of *StSUSIa* (**B**), *StSUSIb* (**C**), *StSUSIc* (**D**), *StSUSII* (**E**), *StSUSIIIa* (**F**), *StSUSIIIb* (**G**), *StSPSI* (**H**), *StSPSII* (**I**), *StSPSIII* (**J**), and *StSPSIV* (**K**), shown over one day at 4 h intervals. The continuous white and gray bars indicate day and night, respectively.

*StSUSIb*, *StSUSIc*, and *StSUSII* exhibited high relative expressions in tissues such as the leaves, flowers, tubers, and petals. *StSPSI* was relatively highly expressed in the *StSPS* gene family members among the roots, shoots, leaves, sepals, and callus tissue (Figure 5A). The relative expression levels of most *StSUS* and *StSPS* genes show significant diurnal fluctuations (Figure 5B–K). The relative expression levels of *StSUSIb*, *StSUSII*, *StSUSIIIa*, and *StSUSIIIb* were significantly higher at night than during the day (Figure 5B–G). The relative expression levels of *StSPSI*, *StSPSII*, and *StSPSIII* were significantly higher during the day than at night (Figure 5H–J). The relative expression levels of *StSPSIV* did not exhibit significant diurnal variations (Figure 5K).

### 3.5. The Expression of StSUS and StSPS Genes under Sugar Treatment

**Figure 6:**
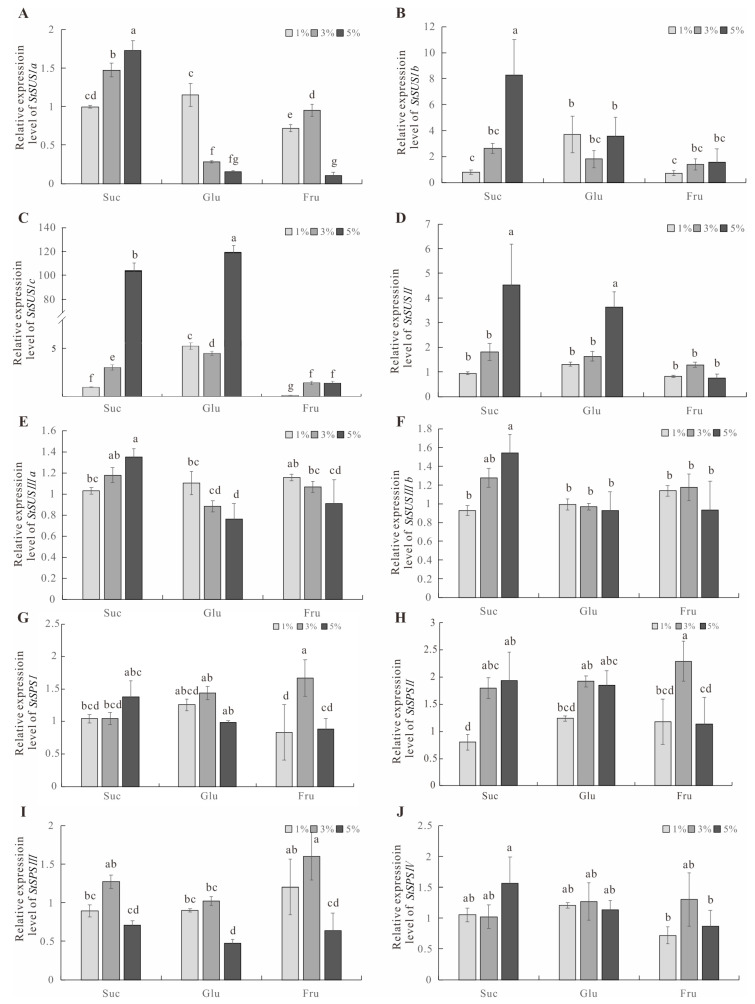
qRT-PCR expression analysis of six *StSUS* and four *StSPS* genes under 1%, 3%, and 5% sugar treatments. (**A**–**J**) The expression levels of the *StSUSs* and *StSPSs* were measured in response to 1%, 3%, and 5% sucrose, glucose, and fructose, respectively, comparing them to the treatment with 1% sucrose. At least three samples were used. Different letters above the columns in the figure indicate significant differences based on Duncan’s test at a *p* < 0.05 significance level.

The *StSUS* family’s gene expression level was up-regulated with increases in the sucrose concentration (Figure 6A–F), especially t he expression level of *StSUSIc*, which increased most obviously. The expression of *StSPSII* in the *StSPS* gene family increased significantly under the 3–5% sucrose treatment, and the expression of the *StSPSI* and *StSPSIV* genes increased under the 5% sucrose condition. The expression levels of *StSUS* family genes showed different trends with increases in the glucose concentration, in which the expression levels of *StSUSIa* and *StSUSIIIa* were inhibited (Figure 6A,E), and the expression levels of *StSUSIc* and *StSUSII* increased (Figure 6C,D). The expression level of *StSPSs* showed no significant change at the 3% glucose concentration, but at the 5% glucose concentration, the expression levels of *StSPSI* and *StSPSIII* were significantly inhibited (Figure 6G–J). The expression of *StSUS* family genes was not significantly changed at the 3% fructose concentration but was down-regulated at the 5% fructose concentration compared with the 1% fructose concentration. The transcript level of the *StSPS* family genes was increased with the 3% fructose treatment. No significant changes were observed under the 5% fructose treatment, except for a down-regulation of the *StSPSIII* gene (Figure 6G–J). 

## 4. Discussion

### 4.1. The Conserved Motifs of StSUS and StSPS Genes and Gene Evolution 

SUS is ubiquitous in higher plant genomes, and the number of homologous genes is generally between five and seven. Different research teams have reported that plant SUS can be divided into three subfamilies: *SUSI*, *SUSII*, and *SUSIII*. *SUSI* can be divided into monocotyledonous and dicotyledonous plants, while *SUSII* and *SUSIII* mainly comprise dicotyledonous plants, except for rice and maize [2,32]. The SUSs in plants were divided into three subfamilies, *SUSI*, *SUSII*, and *SUSIII*, which agreed with a previous report [32,33,34]. Langenkämper et al. [35] divided the SPS family into three subfamilies. These subfamily genes are widely distributed in monocotyledonous and dicotyledonous plants. Studies on wheat and several other plants have divided SPSs into four subfamilies [36,37,38]. The results of this study were consistent with the three-subgroup classifications (Figure 2) [39,40,41]. 

Imbalanced evolution among gene family members has led to functional diversity in the *Gossypium* species [42]. Studies on the *SUS* genes from different species have shown that the evolution of gene family members exhibits a conserved protein structure and amino acid sequence, and differences in gene expression and function [1,2,32]. This study also shows that there are apparent evolutionary differences among *StSUS* gene members (Figure 3B), and that *StSUSIc* has higher expression levels than other family members (Figure 5A). 

### 4.2. Expression of StSUSs and StSPSs Regulates Sucrose Metabolism and Plant Growth

The oxygen content is low in the cells of storage organs with an active energy metabolism, such as in potato tubers, developing seeds, and fruits. Plants need to adjust the sucrose metabolism in their storage organ cells with a high metabolism to maintain normal metabolism, and the SUS pathway, which consumes less oxygen, is preferred over the invertase pathway [16]. SUSs may also participate in other abiotic stress processes. For example, when rice seeds are exposed to high-temperature conditions, an overexpression of the *OsSUS3* gene can reduce the chalkiness of the seeds, reducing the damage of high temperatures to rice yield and quality [9]. Heterologous expression of the potato *StSUS* gene in cotton increases fiber quality and yield [43]. Down-regulation of *CsSUS4* gene expression inhibits the growth of cucumber plants, and their overexpression of *CsSUS4* seedlings produces large flowers and heavy fruits [44]. Overexpression of the *OsSUS3* gene in rice increases starch accumulation and grain weight. SUS affects carbon allocation metabolism and regulates plant growth and development [45]. A recent report showed that sextuple mutants (sus1sus2sus3sus4sus5sus6) exhibit ADPG and starch levels in Arabidopsis similar to those in the wild type [46]. Treatment with 60 mM sucrose induced the up-regulation of the tuber-preferred *SUS* gene *StSUS4* in leaves, and treatment with 200 mM further enhanced its expression, in [1,47]. The transcriptional expression level of the *SPS* gene can affect the activity of the SPS enzyme, thus regulating sucrose content [48]. In the present study, exogenous sucrose treatment induced the gene expression of both *StSUSs* and *StSPSs*, and the relative expression level of the *StSUS1c* gene was the highest (Figure 6). The expression level of *StSUSs* was up-regulated under high concentrations of exogenous sucrose, but high concentrations of exogenous glucose and fructose inhibited the expression of *StSUS* and *StSPS* genes (Figure 6A–J), which agrees with previous reports [1,49] stating that high concentrations of fructose and glucose may inhibit potato seedling growth and development. 

### 4.3. Sucrose Metabolism-Related Genes and the Circadian Rhythm

The circadian clock involves several biological processes, from primary metabolism, hormone signaling, growth, and development to metabolic processes [50,51]. The circadian clock genes, including *StGI*, *StPRR,* and *StEFM*, were significantly differentially expressed in potatoes, indicating that an essential role for the plant circadian pathway exists in regulating tuberization [52]. The circadian clock regulates the transcript levels of several starch biosynthesis-related genes [53,54]. The transcription level of *GBSS1* is regulated by MYB-associated circadian clock-associated 1 and Late Elongated Hypocotyl (LHY) transcription factors in *Arabidopsis* leaves [55], and the circadian fluctuation pattern of the *AGPase* gene is different from that of *GBSS1* [56]. The nitrate reductase gene expression level and enzymatic activity in tobacco leaves shows oscillatory changes [15]. The transcript levels of sucrose synthase genes in potato leaves and tubers showed circadian fluctuations and were associated with starch metabolism [16,25], which suggested that it may be feasible to regulate starch degradation and promote starch synthesis via circadian rhythms. This study found that the gene expression level of the *StSUSs* and *StSPSs* exhibited circadian oscillation, consistent with previous reports [57]. The mechanism of circadian rhythm-mediated expressions of *StSUSs* and *StSPSs* involved in the regulation of sucrose metabolism needs further study.

## 5. Conclusions

We identified six *StSUS* and four *StSPS* genes and analyzed their gene structure, conserved motifs, chromosome position, promoter elements, phylogenetic tree, and tissue-specific expression patterns in potatoes. Gene expression analysis revealed that the expression level of *StSUSIc* was significantly higher than that of other *SUSs* in various tissues. Ka/Ks analysis showed that *StSUSIb* and *StSUSIc* were subject to more-significant homozygous selection pressure. The gene expression of *StSUSs* and *StSPSs* changed significantly under different exogenous sugar applications and circadian rhythms. Our results will promote further study on sucrose metabolism-related gene regulation and manipulation in plants. 

## Figures and Tables

**Table 1:** Basic information on *StSUS* and *StSPS* gene family members in potato reference genome DM 6.1 and DM 8.1.

Gene Name	Gene ID in DM 6.1	Gene ID in DM 8.1	CDS Length (bp)	Protein Length (a.a)	Predicted Mw (kDa)	Theoretical *pI*
*StSUSIa*	Soltu.DM.07G013360	DM8C07G13790	2412	803	91.4	5.88
*StSUSIb*	Soltu.DM.07G013370	DM8C07G13800	2418	805	92.6	6.03
*StSUSIc*	Soltu.DM.12G026390	DM8C12G03720	2418	805	92.5	5.87
*StSUSII*	Soltu.DM.09G031820	DM8C09G32330	2436	811	92.8	6.1
*StSUSIIIa*	Soltu.DM.02G020800	DM8C02G21070	2286	761	86.7	8.53
*StSUSIIIb*	Soltu.DM.03G019120	DM8C03G20010	2679	892	100.7	6.04
*StSPSI*	Soltu.DM.07G003160	DM8C07G03460	3165	1054	118.5	6.39
*StSPSII*	Soltu.DM.08G010240	DM8C08G10890	3120	1039	116.7	6.61
*StSPSIII*	Soltu.DM.09G029230	DM8C09G29940	3195	1064	119.6	6.55
*StSPSIV*	Soltu.DM.11G017190	DM8C11G17260	3102	1033	116.5	6.69

## Data Availability

The potato genome sequences (*Solanum tuberosum* L.) were downloaded from Phytozome (http://www.phytozome.net/ (accessed on 29 March 2021)) and another website (http://www.bioinformaticslab.cn/files/dm8/ (accessed on 25 Augest 2023)). The sequences of SUSs and SPSs were obtained from 13 species, namely *Arabidopsis thaliana*, *Brassica oleracea*, *Brassica rapa*, *Cucumis sativus*, *Glycine max*, *Malus domestica*, *Musa acuminata*, *Oryza sativa*, *Setaria italica*, *Solanum lycopersicum*, *Solanum tuberosum*, *Triticum aestivum*, and *Zea mays*, collected from Phytozome (https://phytozome.jgi.doe.gov/pz/portal.html (accessed on 21 April 2022) and NCBI (www.ncbi.nlm.nih.gov (accessed on 28 April 2022). For tissue-specific gene expression, we used DM_1–3_516_R44_potato.v6.1.TPM_gene_expression_matrix (http://spuddb.uga.edu/ (accessed on 18 December 2022). Our collection of potato materials was permitted and complied with relevant institutional, national, and international guidelines and legislation. The materials in this study are available from the corresponding author upon request.

## Supplementary Materials

The following supporting information can be downloaded at: https://www.mdpi.com/article/10.3390/metabo14010070/s1, Table S1: The primers used in this study. Table S2. Variance analysis of the relative expression levels of *StSUS* and *StSPS* genes under 1%, 3%, and 5% sucrose, glucose, and fructose treatments.

## References

[1] Yoon J., Cho L.H., Tun W., Jeon J.S., An G. (2021). Sucrose signaling in higher plants. Plant Sci..

[2] Stein O., Granot D. (2019). An Overview of Sucrose Synthases in Plants. Front. Plant Sci..

[3] Bahaji A., Li J., Sánchez-López Á.M., Baroja-Fernández E., Muñoz F.J., Ovecka M., Almagro G., Montero M., Ezquer I., Etxeberria E. (2014). Starch biosynthesis, its regulation and biotechnological approaches to improve crop yields. Biotechnol. Adv..

[4] Zrenner R., Salanoubat M., Willmitzer L., Sonnewald U. (1995). Evidence of the crucial role of sucrose synthase for sink strength using transgenic potato plants (*Solanum tuberosum* L.). Plant J..

[5] Baroja-Fernández E., Muñoz F.J., Montero M., Etxeberria E., Sesma M.T., Ovecka M., Bahaji A., Ezquer I., Li J., Prat S. (2009). Enhancing Sucrose Synthase Activity in Transgenic Potato (*Solanum tuberosum* L.) Tubers Results in Increased Levels of Starch, ADPglucose and UDPglucose and Total Yield. Plant Cell Physiol..

[6] Li J., Baroja-Fernández E., Bahaji A., Muñoz F.J., Ovecka M., Montero M., Sesma M.T., Alonso-Casajús N., Almagro G., Sánchez-López A.M. (2013). Enhancing Sucrose Synthase Activity Results in Increased Levels of Starch and ADP-Glucose in Maize (*Zea mays* L.) Seed Endosperms. Plant Cell Physiol..

[7] Baroja-Fernández E., Muñoz F.J., Li J., Bahaji A., Almagro G., Montero M., Etxeberria E., Hidalgo M., Sesma M.T., Pozueta-Romero J. (2012). Sucrose synthase activity in the sus1/sus2/sus3/sus4 Arabidopsis mutant is sufficient to support normal cellulose and starch production. Proc. Natl. Acad. Sci. USA.

[8] Zhao C., Hua L.N., Liu X.F., Li Y.Z., Shen Y.Y., Guo J.X. (2017). Sucrose synthase FaSS1 plays an important role in the regulation of strawberry fruit ripening. Plant Growth Regul..

[9] Takehara K., Murata K., Yamaguchi T., Yamaguchi K., Chaya G., Kido S., Iwasaki Y., Ogiwara H., Ebitani T., Miura K. (2018). Thermo-responsive allele of sucrose synthase 3 (*Sus3*) provides high-temperature tolerance during the ripening stage in rice (*Oryza sativa* L.). Breed. Sci..

[10] Chen S., Hajirezaei M., Börnke F. (2005). Differential Expression of Sucrose-Phosphate Synthase Isoenzymes in Tobacco Reflects Their Functional Specialization during Dark-Governed Starch Mobilization in Source Leaves. Plant Physiol..

[11] Okamura M., Aoki N., Hirose T., Yonekura M., Ohto C., Ohsugi R. (2011). Tissue specificity and diurnal change in gene expression of the sucrose phosphate synthase gene family in rice. Plant Sci..

[12] Zhang Y., Zeng D., Liu Y., Zhu W. (2022). *SlSPS*, a Sucrose Phosphate Synthase Gene, Mediates Plant Growth and Thermotolerance in Tomato. Horticulturae.

[13] Anur R.M., Mufithah N., Sawitri W.D., Sakakibara H., Sugiharto B. (2020). Overexpression of Sucrose Phosphate Synthase Enhanced Sucrose Content and Biomass Production in Transgenic Sugarcane. Plants.

[14] Cai C., Liu S., Liu J., Wen H., Li L., Wang Q., Li L., Wang X. (2023). Screening and Identification of Potato StSPS1, a Potential Crucial Gene Regulating Seed Potato Vigor. Horticulturae.

[15] Farré E.M., Weise S.E. (2012). The interactions between the circadian clock and primary metabolism. Curr. Opin. Plant Biol..

[16] Geigenberger P., Stitt M. (2000). Diurnal changes in sucrose, nucleotides, starch synthesis and AGPS transcript in growing potato tubers that are suppressed by decreased expression of sucrose phosphate synthase. Plant J..

[17] Yonekura M., Aoki N., Hirose T., Onai K., Ishiura M., Okamura M., Ohsugi R., Ohto C. (2013). The promoter activities of sucrose phosphate synthase genes in rice, *OsSPS1* and *OsSPS11*, are controlled by light and circadian clock, but not by sucrose. Front. Plant Sci..

[18] Fiorucci A.S., Galvão V.C., Ince Y.Ç., Boccaccini A., Goyal A., Allenbach Petrolati L., Trevisan M., Fankhauser C. (2020). PHYTOCHROME INTERACTING FACTOR 7 is important for early responses to elevated temperature in Arabidopsis seedlings. New Phytol..

[19] Haydon M.J., Mielczarek O., Frank A., Román Á., Webb A.A.R. (2017). Sucrose and Ethylene Signaling Interact to Modulate the Circadian Clock. Plant Physiol..

[20] Yang X., Zhang L., Guo X., Xu J., Zhang K., Yang Y., Yang Y., Jian Y., Dong D., Huang S. (2023). The gap-free potato genome assembly reveals large tandem gene clusters of agronomical importance in highly repeated genomic regions. Mol. Plant.

[21] Pham G.M., Hamilton J.P., Wood J.C., Burke J.T., Zhao H., Vaillancourt B., Ou S., Jiang J., Buell C.R. (2020). Construction of a chromosome-scale long-read reference genome assembly for potato. GigaScience.

[22] Hoopes G., Meng X., Hamilton J.P., Achakkagari S.R., de Alves Freitas Guesdes F., Bolger M.E., Coombs J.J., Esselink D., Kaiser N.R., Kodde L. (2022). Phased, chromosome-scale genome assemblies of tetraploid potato reveal a complex genome, transcriptome, and predicted proteome landscape underpinning genetic diversity. Mol. Plant.

[23] Bao Z., Li C., Li G., Wang P., Peng Z., Cheng L., Li H., Zhang Z., Li Y., Huang W. (2022). Genome architecture and tetrasomic inheritance of autotetraploid potato. Mol. Plant.

[24] Li M., Xie H., He M., Su W., Yang Y., Wang J., Ye G., Zhou Y. (2020). Genome-wide identification and expression analysis of the *StSWEET* family genes in potato (*Solanum tuberosum* L.). Genes. Genom..

[25] Xu Y., Wang Y., Mattson N., Yang L., Jin Q. (2017). Genome-wide analysis of the *Solanum tuberosum* (potato) trehalose-6-phosphate synthase (TPS) gene family: Evolution and differential expression during development and stress. BMC Genom..

[26] Li Y., Liang J., Zeng X., Guo H., Luo Y., Kear P., Zhang S., Zhu G. (2021). Genome-wide Analysis of MYB Gene Family in Potato Provides Insights into Tissue-specific Regulation of Anthocyanin Biosynthesis. Hortic. Plant J..

[27] He F., Duan S., Jian Y., Xu J., Hu J., Zhang Z., Lin T., Cheng F., Li G. (2022). Genome-wide identification and gene expression analysis of the 14-3-3 gene family in potato (*Solanum tuberosum* L.). BMC Genom..

[28] Kumar S., Stecher G., Li M., Knyaz C., Tamura K. (2018). MEGA X: Molecular Evolutionary Genetics Analysis across Computing Platforms. Mol. Biol. Evol..

[29] Wang Y., Tang H., DeBarry J.D., Tan X., Li J., Wang X., Lee T., Jin H., Marler B., Guo H. (2012). MCScanX: A toolkit for detection and evolutionary analysis of gene synteny and collinearity. Nucleic Acids Res..

[30] Wang D., Zhang Y., Zhang Z., Zhu J., Yu J. (2010). KaKs_Calculator 2.0: A Toolkit Incorporating Gamma-Series Methods and Sliding Window Strategies. Genom. Proteom. Bioinf.

[31] Chen C., Chen H., Zhang Y., Thomas H.R., Frank M.H., He Y., Xia R. (2020). TBtools: An Integrative Toolkit Developed for Interactive Analyses of Big Biological Data. Mol. Plant.

[32] Xu X., Yang Y., Liu C., Sun Y., Zhang T., Hou M., Huang S., Yuan H. (2019). The evolutionary history of the sucrose synthase gene family in higher plants. BMC Plant Biol..

[33] Jiang Z., Zhang H., Gao S., Zhai H., He S., Zhao N., Liu Q. (2023). Genome-Wide Identification and Expression Analysis of the Sucrose Synthase Gene Family in Sweet Potato and Its Two Diploid Relatives. Int. J. Mol. Sci..

[34] Wang D., Zhao J., Qin Y., Qin Y., Hu G. (2021). Molecular cloning, characterization and expression profile of the sucrose synthase gene family in *Litchi chinensis*. Hortic. Plant J..

[35] Langenkämper G., Fung R.W.M., Newcomb R.D., Atkinson R.G., Gardner R.C., MacRae E.A. (2002). Sucrose Phosphate Synthase Genes in Plants Belong to Three Different Families. J. Mol. Evol..

[36] Castleden C.K., Aoki N., Gillespie V.J., MacRae E.A., Quick W.P., Buchner P., Foyer C.H., Furbank R.T., Lunn J.E. (2004). Evolution and Function of the Sucrose-Phosphate Synthase Gene Families in Wheat and Other Grasses. Plant Physiol..

[37] Duan Y., Yang L., Zhu H., Zhou J., Sun H., Gong H. (2021). Structure and Expression Analysis of Sucrose Phosphate Synthase, Sucrose Synthase and Invertase Gene Families in *Solanum lycopersicum*. Int. J. Mol. Sci..

[38] Ma P., Zhang X., Chen L., Zhao Q., Zhang Q., Hua X., Wang Z., Tang H., Yu Q., Zhang M. (2020). Comparative analysis of sucrose phosphate synthase (*SPS*) gene family between *Saccharum officinarum* and *Saccharum spontaneum*. BMC Plant Biol..

[39] Zhang L., Zhu L., Xu Y., Lü L., Li X., Li W., Liu W., Ma F., Li M., Han D. (2023). Genome-wide identification and function analysis of the sucrose phosphate synthase *MdSPS* gene family in apple. J. Integr. Agr..

[40] Li M., He Q., Huang Y., Luo Y., Zhang Y., Chen Q., Wang Y., Lin Y., Zhang Y., Liu Z. (2021). Sucrose synthase gene family in *Brassica juncea*: Genomic organization, evolutionary comparisons, and expression regulation. PeerJ.

[41] Huang T., Luo X., Fan Z., Yang Y., Wan W. (2021). Genome-wide identification and analysis of the sucrose synthase gene family in cassava (*Manihot esculenta Crantz*). Gene.

[42] Ge Q., Cui Y., Li J., Gong J., Lu Q., Li P., Shi Y., Shang H., Liu A., Deng X. (2020). Disequilibrium evolution of the Fructose-1,6-bisphosphatase gene family leads to their functional biodiversity in *Gossypium species*. BMC Genom..

[43] Ahmed M., Iqbal A., Latif A., Din S.u., Sarwar M.B., Wang X., Rao A.Q., Husnain T., Ali Shahid A. (2020). Overexpression of a Sucrose Synthase Gene Indirectly Improves Cotton Fiber Quality Through Sucrose Cleavage. Front. Plant Sci..

[44] Fan J., Wang H., Li X., Sui X., Zhang Z. (2018). Down-Regulating Cucumber Sucrose Synthase 4 (*CsSUS4*) Suppresses the Growth and Development of Flowers and Fruits. Plant Cell Physiol..

[45] Dominguez P.G., Donev E., Derba-Maceluch M., Bünder A., Hedenström M., Tomášková I., Mellerowicz E.J., Niittylä T. (2021). Sucrose synthase determines carbon allocation in developing wood and alters carbon flow at the whole tree level in aspen. New Phytol..

[46] Fünfgeld M.M.F.F., Wang W., Ishihara H., Arrivault S., Feil R., Smith A.M., Stitt M., Lunn J.E., Niittylä T. (2022). Sucrose synthases are not involved in starch synthesis in Arabidopsis leaves. Nature Plants.

[47] Fu H., Park W.D. (1995). Sink- and vascular-associated sucrose synthase functions are encoded by different gene classes in potato. Plant Cell.

[48] Verma A.K., Upadhyay S.K., Verma P.C., Solomon S., Singh S.B. (2011). Functional analysis of sucrose phosphate synthase (*SPS*) and sucrose synthase (*SS*) in sugarcane (*Saccharum*) cultivars. Plant Biol..

[49] Gibson S.I. (2005). Control of plant development and gene expression by sugar signaling. Curr. Opin. Plant Biol..

[50] Venkat A., Muneer S. (2022). Role of Circadian Rhythms in Major Plant Metabolic and Signaling Pathways. Front. Plant Sci..

[51] Kim J.A., Kim H.-S., Choi S.-H., Jang J.-Y., Jeong M.-J., Lee S.I. (2017). The Importance of the Circadian Clock in Regulating Plant Metabolism. Int. J. Mol. Sci..

[52] Niu Y., Li G., Jian Y., Duan S., Liu J., Xu J., Jin L. (2022). Genes related to circadian rhythm are involved in regulating tuberization time in potato. Hortic. Plant J..

[53] Kötting O., Kossmann J., Zeeman S.C., Lloyd J.R. (2010). Regulation of starch metabolism: The age of enlightenment?. Curr. Opin. Plant Biol..

[54] Lu Y., Gehan J.P., Sharkey T.D. (2005). Daylength and Circadian Effects on Starch Degradation and Maltose Metabolism. Plant Physiol..

[55] Tenorio G., Orea A., Romero J.M., Mérida Á. (2003). Oscillation of mRNA level and activity of granule-bound starch synthase I in Arabidopsis leaves during the day/night cycle. Plant Mol. Biol..

[56] Wang S.J., Yeh K.W., Tsai C.Y. (2001). Regulation of starch granule-bound starch synthase I gene expression by circadian clock and sucrose in the source tissue of sweet potato. Plant Sci..

[57] Liu L., Zheng J. (2022). Identification and expression analysis of the sucrose synthase gene family in pomegranate (*Punica granatum* L.). PeerJ.

