# Supplementary material for: Phylogenetic and Expression Analysis of the Sucrose Synthase and Sucrose Phosphate Synthase Gene Family in Potatoes
Source: Metabolites. 2024 Jan 20;14(1):70. doi: 10.3390/metabo14010070 (PMC10820854; doi:10.3390/metabo14010070)
Supplement: Supplementary file 1 [file metabolites-14-00070-s001.zip › metabolites-2795367-supplementary.pdf]

Table S1. The primers used in this study.

Genes	Forward	Reverse	Melting Temperature
StSPS Ⅰ	GTTTATTGTCAAAATGGGAG	ATTCCTCGTCCGCTTGGATG	84℃
StSPS Ⅱ	GCAATATATTGCCAAAGCGG	CTCAAGAGGGTAGTTCCTGT	83℃
*StSPS*Ⅲ	ATGTATTGCAGGAATTCAAC	TTCTCTTAGGTAGCTCCCT	84℃
*StSPS*Ⅳ	TGTCTACACACATGCTGCA	GTTGAAGCTGTCTTCATTG	84℃
StSUS Ⅰ *a*	TCCAAAGAAGATCCAAGTT	AACTTTCTCAGCAGATTTC	82℃
StSUS Ⅰ *b*	TTGAGAAATGTAAGGTAGA	CAAGTTGAGCCAGCTTGC	84℃
StSUS Ⅰ *c*	AAATGCAAGAAAGATCCTTC	ACAGCTTCAGCCATCTTAC	84℃
StSUS Ⅱ	CCAACGATGCGAACAAAAT	GAACAGATTTTACCAACTC	83℃
StSUSIIIa	ATGGAAGATCTATGCAAAC	**ATGACTGGCTGTTCGATGA**	84℃
StSUSIIIb	ATGAAGAGCCCTTGCCAGCT	TCATTCAACTATACGGTACA	84℃
ElF3e	GGAGCACAGGAGAAGATGAAGGAG	CGTTGGTGAATGCGGCAGTAGG	83℃

Table S2. Variance analysis of the relative expression level of the *StSUSs* and *StSPSs* genes under 1%, 3%, and 5% sucrose, glucose and fructose treatments.

	Source of variation	SS	df	MS	F	P-value
SUS Ⅰa	Treatment	8.02	8	1.00	105.79	0.00
	Error	0.17	18	0.01		
SUS Ⅰb	Treatment	132.73	8	16.59	7.48	0.00
	Error	39.92	18	2.22		
SUS Ⅰc	Treatment	54781.52	8	6847.69	379.42	0.00
	Error	324.86	18	18.05		
SUS Ⅱ	Treatment	42.16	8	5.27	9.41	0.00
	Error	10.08	18	0.56		
SUSⅢa	Treatment	0.76	8	0.09	5.48	0.00
	Error	0.31	18	0.02		
SUSⅢb	Treatment	1.06	8	0.13	3.75	0.01
	Error	0.63	18	0.04		
SPS Ⅰ	Treatment	1.99	8	0.25	3.89	0.01
	Error	1.15	18	0.06		
SPS Ⅱ	Treatment	5.87	8	0.73	4.50	0.00
	Error	2.93	18	0.16		
SPSⅢ	Treatment	2.94	8	0.37	7.33	0.00
	Error	0.90	18	0.05		
SPSⅣ	Treatment	1.48	8	0.18	1.77	0.15
	Error	1.88	18	0.10		
